# Supplementary material for: A Global Screen for Assembly State Changes of the Mitotic Proteome by SEC-SWATH-MS
Source: Cell Syst. 2020 Feb 26;10(2):133–155.e6. doi: 10.1016/j.cels.2020.01.001 (PMC7042714; doi:10.1016/j.cels.2020.01.001)

O75164 | KDM4A_HUMAN | KDM4A JHDM3A JMJD2 JMJD2A KIAA0677
Monomer MW [kDa]: 120.662 Monomer expected elution fraction: 39

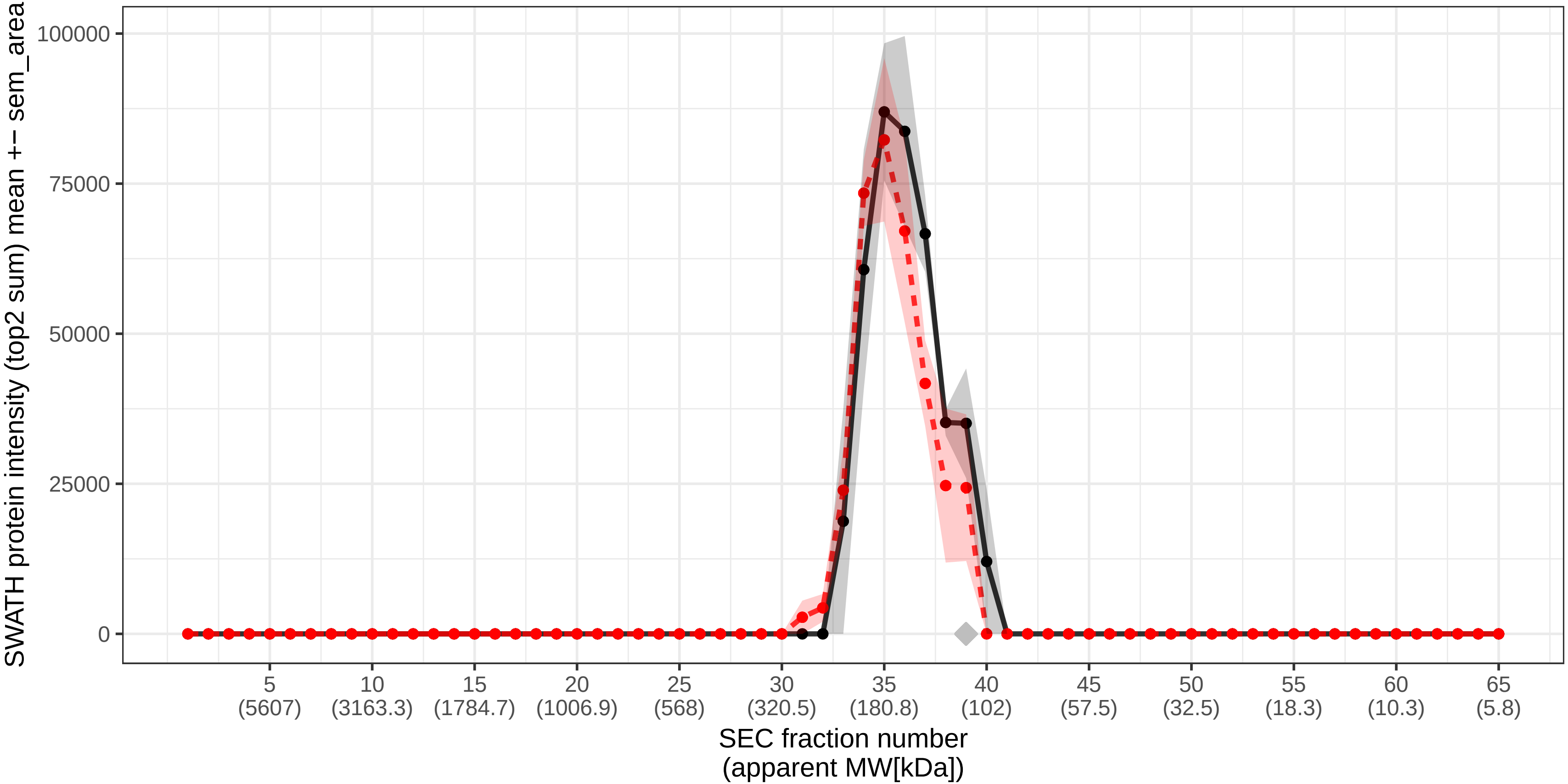

Supplement: Data S1. SEC-SWATH-MS Protein Chromatograms, Related to Figure 1 [file mmc6.zip › SECchrom_O75164_KDM4A_HUMAN_KDM4A_JHDM3A_JMJD2_JMJD2A_KIAA0.pdf]
